# Hydrated Calcium Silicate Erosion in Sulfate Environments a Molecular Dynamics Simulation Study

**DOI:** 10.3390/ma17236005

**Published:** 2024-12-07

**Authors:** Mengjie You, Xiaosan Yin, Yuzhou Sun, Hairong Wu, Jimin Li, Xiangming Zhou

**Affiliations:** 1 School of Architecture and Engineering, Zhongyuan University of Technology, Zhengzhou 451197, China; 15093638665@163.com (M.Y.); yinxiaosan_3@126.com (X.Y.); li3135752022@163.com (J.L.); 2 Henan Mechanics and Engineering Structures Engineering Research Center, Zhengzhou 451197, China; 3 School of Civil and Transportation Engineering, Henan University of Urban Construction, Pingdingshan 467041, China; zjuwhr@huuc.edu.cn; 4 Department of Civil and Environmental Engineering, Brunel University London, London UB8 3PH, UK; xiangming.zhou@brunel.ac.uk

**Keywords:** hydrated calcium silicate, sodium sulfate erosion, molecular dynamics, micro-mechanisms

## Abstract

To investigate the micro-mechanism of the erosion of hydrated calcium silicate (C-S-H gel) in a sulfate environment, a solid–liquid molecular dynamics model of C-S-H gel/sodium sulfate was developed. This model employs molecular dynamics methods to simulate the transport processes between C-S-H gel and corrosive ions at concentrations of 5%, 8%, and 10% sodium sulfate (Na_2_SO_4_), aiming to elucidate the interaction mechanism between sulfate and C-S-H gel. The micro-morphology of the eroded samples was also investigated using scanning electron microscopy (SEM). The findings indicate that the adsorption capacity of C-S-H for ions significantly increases with higher concentrations of Na_2_SO_4_ solution. Notably, the presence of sulfate ions facilitates the decalcification reaction of C-S-H, leading to the formation of swollen gypsum and AFt (ettringite). This process results not only in the hydrolysis of the C-S-H gel but also in an increase in the diffusion coefficients of Na^+^ and Ca^2+^, thereby exacerbating the erosion. Additionally, the pore surfaces of the C-S-H structure exhibited strong adsorption of Na^+^, and as the concentration of Na_2_SO_4_ solution increased, Na+ was more stably adsorbed onto the C-S-H pore surfaces via Na-O_s_ bonds. The root-mean-square displacement curves of water molecules were significantly higher than those of ${\mathrm{SO}}_{4}^{2-}$, Na^+^ and Ca^2+^, which indicated that ${\mathrm{SO}}_{4}^{2-}$ could co-penetrate and migrate with water molecules faster compared with other ions in the solution containing ${\mathrm{SO}}_{4}^{2-}$, resulting in stronger corrosion and hydrolysis effects on the C-S-H structure.

## 1. Introduction

Sulfate erosion is a prevalent form of corrosion affecting concrete structures, particularly in coastal industrial cities, western salt lakes, and similar regions. The damage inflicted on concrete by sulfate solutions can be attributed to two primary mechanisms: physical and chemical. Physical damage manifests as the penetration of sulfate solutions into the concrete, which induces the formation of expansive crystals. This process not only initiates the formation of cracks but also exacerbates their growth. In contrast, chemical damage arises from the interaction between the sulfate solution and the hydration products of the cement within the concrete, resulting in the generation of expansive substances such as AFt (ettringite) and CaSO_4_·2H_2_O in the cracked regions [1]. Specifically, in an environment containing Na_2_SO_4_, inadequately hydrated cement particles react with the components of the solution to produce new hydration products. These products tend to accumulate and expand within the cracks, with both their size and quantity significantly increasing over time and with rising Na_2_SO_4_ concentrations. To some extent, these newly formed hydration products can contribute to the self-healing of the cracks. However, when the concentration of Na_2_SO_4_ exceeds a critical threshold, Can lead to certain products produced in hydration reactions (e.g., ettringite and gypsum) that can significantly disrupt the internal bond of the concrete. This expansion can create significant crystallization pressure, which can lead to secondary cracking. Calcite and gypsum formation is due to sulfate attack, a chemical reaction between sulfate and certain cement hydration phases, which further exacerbates material deterioration [2]. Therefore, understanding the deterioration mechanisms of sulfate attack on concrete at the microscopic level is essential for mitigating its effects on concrete structures.

Molecular Dynamics Method (MD) is one of the most important research methods to study the microscopic mechanism of sulfate erosion of concrete and ion distribution. Yang et al. [3] used a novel C-S-H gel pore model to analyze the microscopic mechanism of salt crystallization in cementitious materials and found that the depth of water molecules entering the C-S-H gel pores in NaCl solution varied parabolically with time, followed by frequent penetration of Na^+^ and Cl^−^, whereas the transport rate of ${\mathrm{SO}}_{4}^{2-}$ was significantly lower than that of Cl^−^ in Na_2_SO_4_ solution. With increasing temperature, the water transport rate in the nano-C-S-H channels accelerated, but the Na^+^ and ${\mathrm{SO}}_{4}^{2-}$ transport rates decreased significantly, which was mainly attributed to the immobilization of the C-S-H surface and the adsorption effect of the ionic clusters [4]. On the C-S-H surface, the composition of the hydration shell (including adsorbed water molecules, surface Ca^2+^, and silicate oxygen sites) limits the ion transport capacity, leading to prolonged hydration shell exchange time for Na^+^ and ${\mathrm{SO}}_{4}^{2-}$ adsorbed on the C-S-H surface and enhanced stability of the ion clusters [5]. The attraction of Ca^2+^ to ${\mathrm{SO}}_{4}^{2-}$ on C-S-H surfaces with higher Ca/Si ratios enhanced anion immobilization [6]. In addition, in the nanopores of C-S-H gels, divalent anions are more likely to form strong ion pairs, thus inhibiting overall transport [7]. Although there is less mutual interference between Cl^−^ and ${\mathrm{SO}}_{4}^{2-}$, the coexistence of ${\mathrm{SO}}_{4}^{2-}$ and ${\mathrm{NO}}_{2}^{-}$ significantly reduces the overall transport rate of the solution, especially the transport rate of ${\mathrm{NO}}_{2}^{-}$ decreases dramatically [8]. External harmful ions such as Cl^−^ lead to the hydrolysis of the C-S-H gel, which reduces the strength of the concrete, whereas the addition of water enhances the interaction of Ca^2+^ in C-S-H with the oxygen atoms in the water but does not affect the interaction of Ca^2+^ in the silica chains with the oxygen atoms [9]. Due to the hydrophilic nature of the C-S-H structure, water molecules will accumulate on the pore surface when diffusing in small pore sizes, forming a meniscus-shaped interface, which promotes the strong adsorption of Na^+^ by the C-S-H structure. With the internal reaction of concrete, the generation of fine cracks further promotes the diffusion of Cl^−^ and reduces the mechanical properties of C-S-H. In summary, the study of chloride transport mechanism in concrete revealed the effect of salt solution on C-S-H gel and its mechanism of action in concrete erosion [10].

The current research trend focuses on the adsorption and diffusion behavior of Cl^−^ with the C-S-H structure, while the analysis on the interaction between C-S-H and Na_2_SO_4_ is still insufficient. Molecular dynamics simulations in a sulfate environment are required for a comprehensive understanding of the complex interaction mechanisms between the components of Na_2_SO_4_ and the C-S-H interface. The interactions between Na_2_SO_4_ and C-S-H occur at the atomic and molecular scales, and it is difficult for conventional experimental methods to resolve these microscopic interactions with sufficient precision. Molecular dynamics simulations are able to reveal the distribution of ions on the C-S-H surface, their coordination environment, and their binding modes at the atomic level, and this detailed resolution is essential for an in-depth understanding of the underlying mechanisms of these interactions.

In this study, the migration and adsorption behaviors of ${\mathrm{SO}}_{4}^{2-}$ and Na^+^ in the C-S-H nanopores are systematically investigated by molecular dynamics simulations using the C-S-H/Na_2_SO_4_ model, which in turn analyzes the erosive effect of these processes on the structure of the C-S-H effect. During the simulation process, the researchers collected a series of key data, such as dynamic trajectories, mean square displacements (MSD), and ion concentration changes of the system. Based on these simulation results, the differences in the C-S-H erosion effect and its reaction mechanism between the multi-ion and single-ion environments were further analyzed, revealing the details of the dynamic processes such as physical adsorption, ion substitution, and migration. This provides a solid theoretical foundation for an in-depth understanding of the behavior of C-S-H in sulfate environments.

## 2. Tests and Methods

### 2.1. Concrete Specimen Production

The specimen size is 150 mm × 150 mm × 150 mm, after the production is completed and put into the maintenance room for 28 d, after the test is qualified, the specimen will be immersed in the 0%, 5%, and 8% concentrations of Na_2_SO_4_ solution; the immersion periods are 0, 30, 60, and 90 d, respectively. In order to maintain the concentration of the solution, the solution is replaced at the 50th d. The test is carried out in the same way as the specimen.

### 2.2. SEM Test Methods

The crushed concrete specimens were immersed in anhydrous ethanol for 2 d and then dried in a 45 °C drying oven for 3 d in preparation for microscopic observation.

In order to deeply explore the mechanism of C-S-H erosion under the action of Na_2_SO_4_, microscopic observation (SEM) was carried out on the samples with different corrosion durations, and the samples were selected to be unimmersed (GS1-0), immersed in 8% Na_2_SO_4_ solution for 30 d (GS1-8-30), immersed in 8% Na_2_SO_4_ solution for 60d (GS1-8-60), immersed in water for 90 d (GS1-W-90), 90 d immersion in 5% Na_2_SO_4_ solution (GS1-5-90), and 90 d immersion in 8% Na_2_SO_4_ solution (GS1-8-90). Samples were subjected to scanning electron microscopy.

## 3. Molecular Dynamics Simulation

### 3.1. Molecular Modeling of C-S-H Gels

In this paper, we utilize Materials Studio (2019) software to construct molecular dynamics and solid–liquid models of calcium silicate hydrate (C-S-H) gel, aiming to simulate the erosion process of sodium sulfate on the calcium silicate hydrate model at varying concentrations.

**Figure 1:**
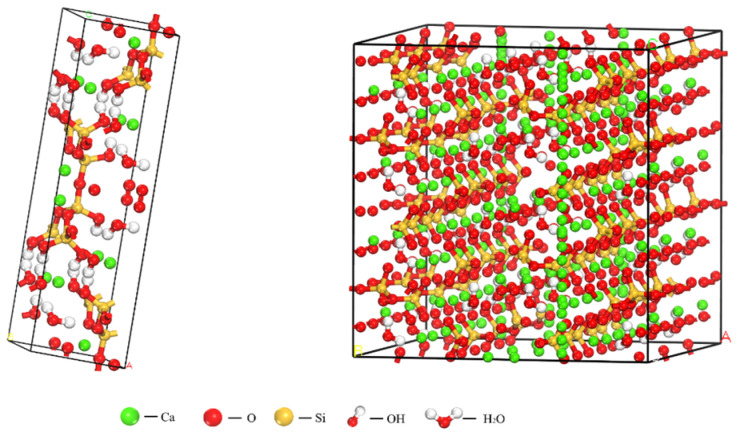
Initial structure of Tobermorite and optimized crystal models.

The chemical compositions of Tobermorite and C-S-H gels exhibit a high degree of similarity to the lamellar structure [11,12]. Therefore, the microscopic initial model of Tobermorite crystals [13] was employed. As illustrated in Figure 1., based on the foundational research conducted by Pellenq et al. [14], a molecular structure model of C-S-H gel (with a Ca/Si ratio of 1.7 and a density of 2.45 g/cm^3^) has been established in accordance with experimental data. To mitigate boundary effects, periodic boundary conditions are implemented in this study.

In the modeling process, the hydroxyl group was first removed from the 11 Å Tobermorite crystal model, followed by supercell construction (4a × 3b × 1c). Due to the higher symmetry of orthorhombic crystals, this approach yielded greater computational efficiency compared to monoclinic crystals, resulting in more accurate computational outcomes and facilitating easier software processing. This is advantageous for the integration of the model results in subsequent stages and data analysis [15]. Therefore, after supercell construction, the model was converted into an orthorhombic crystal structure. The parameters of the orthogonal crystal structure model box are a = 22.11 Å, b = 22.68 Å, c = 23.47 Å, and α = β = γ = 90°. Subsequently, some SiO_2_ and Si_2_O_5_ groups along the silicon chains within the model were randomly deleted to satisfy the Q_n_ distribution, where Q_0_ = 10%, Q_1_ = 64.4%, Q_2_ = 25.6%, Ca/Si = 1.67, and the density was 2.47 g/cm^3^. Finally, the C-S-H model was structurally optimized to achieve the lowest total energy of the system for the stabilized C-S-H structure.

Molecular force fields are typically selected based on the nature and type of material, including the types of intermolecular forces, as well as chemical and hydrogen bonding, which are essential for generating the driving forces among different atoms or molecules. The non-reactive force fields currently employed for molecular dynamics simulations of cement can be categorized into two main groups: general force fields with broad applicability (e.g., COMPASS) and force fields optimized for specific systems (e.g., ClayFF). The COMPASS force field was chosen for this simulation due to its capability to effectively model a wide range of cementitious materials, with calculated structural-mechanical properties that closely align with experimental data [16]. Consequently, this paper selects COMPASS as the simulation force field.

**Figure 2:**
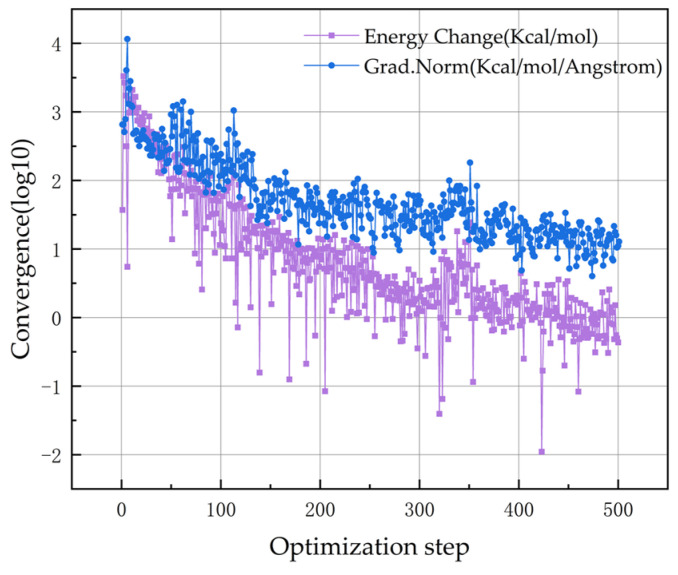
Convergence curve of the C-S-H model.

The structural optimization process consists of two main steps: energy minimization and dynamics optimization. Initially, the C-S-H model undergoes energy minimization, which is performed using the Geometry Optimization option within the Forcite module, employing the COMPASS II force field. The convergence diagram, illustrating the results of the energy minimization, is presented in Figure 2.

**Figure 3:**
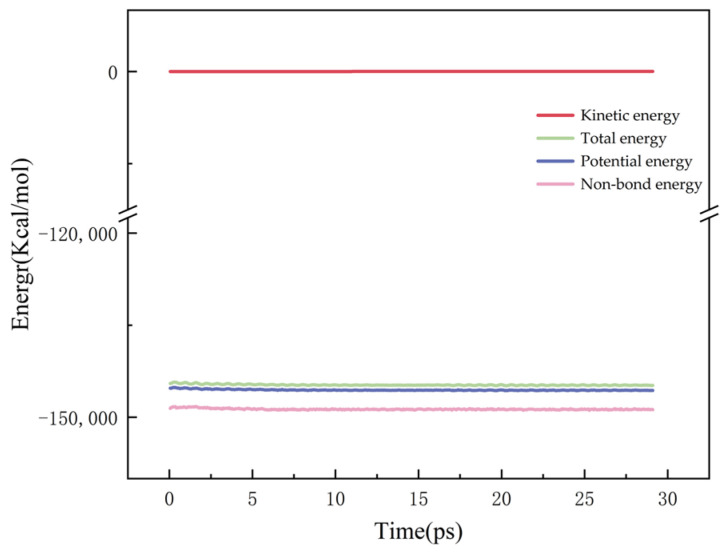
Energy profile after dynamics optimization.

Subsequently, molecular dynamics optimization is conducted on the geometrically optimized model. The dynamics optimization is executed under dynamic conditions, utilizing the COMPASS II force field. The system is synthesized under Constant Temperature and Constant Volume (NVT) conditions, where N represents the number of particles, V denotes the volume of the system, and T indicates the temperature, which is maintained at 298 K. The total optimization duration is set to 50 ps, with a step size of 1 fs. The energy profile resulting from the dynamics optimization is illustrated in Figure 3.

### 3.2. Solid–Liquid Model

**Figure 4:**
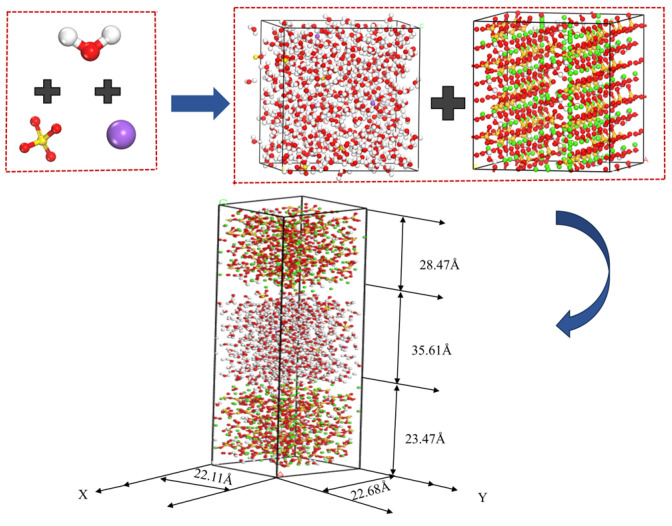
C-S-H/Na_2_SO_4_ solid–liquid modeling process.

A Na_2_SO_4_ solution with concentrations of 5%, 8%, and 10% was selected as the primary research subject and accurately aligned with the C-S-H structure to construct a composite model, as illustrated in Figure 4. In this model, ${\mathrm{SO}}_{4}^{2-}$ and water molecules were randomly and uniformly distributed within the nanopore structure of C-S-H to simulate the actual environmental conditions. The analysis focused on the effects of ${\mathrm{SO}}_{4}^{2-}$ on the water molecules, as well as the transport properties of key ions, such as Ca^2+^, within the pore at varying concentrations. The parameters of the C-S-H model are presented in Table 1.

**Figure 5:**
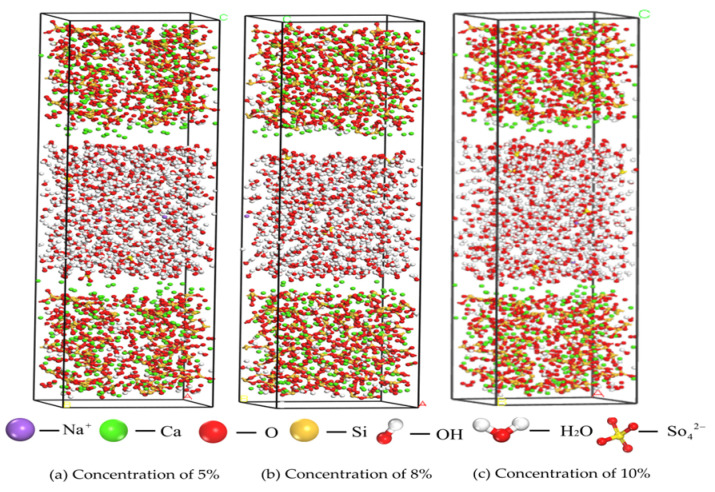
Solid–liquid ionic model.

According to the experimental guidelines established by Minet [17], the pore size range of C-S-H gels was defined to be between 0.5 and 10 nm. Consequently, the interlayer distance in the constructed model was set to 3 nm to ensure the accuracy and validity of the simulation. A cutting tool was employed to adjust the dimensions of the Na_2_SO_4_ solution model to align with the predetermined conditions. Following this, the modified Na_2_SO_4_ solution model was incorporated between the two layers of the supercell model to accurately represent the structure of the interlayer pores within the C-S-H gel, thereby creating a composite model with interlayer characteristics, as illustrated in Figure 5. To effectively mitigate the potential influence of the cyclic structure on the simulation results, a vacuum layer with a thickness of 0.5 nm was introduced between the sulfate solution and the C-S-H model. It helps to ensure that the boundaries of the system do not interfere unnecessarily with the simulation results.

To ensure the stability of the solid–liquid model, an energy minimization optimization process was implemented for the molecular structure following the construction of the concentration distribution model. Intelligent algorithms were selected for this optimization process, and the COMPASS II force field was chosen to guarantee the accuracy and applicability of the simulation. Meanwhile, in order to enhance the optimization efficiency, a partial constraint is adopted, i.e., the positions of all atoms above the Ca sandwich are fixed, while all atoms below the Ca sandwich and inside its C-S-H are allowed to adjust the relaxation of the degrees of freedom. For electrostatic interactions, an atom-based direct summation method was utilized to accurately calculate the interaction forces between charges. In contrast, the Ewald summation method was adopted for van der Waals forces to effectively address long-range interactions and ensure the accuracy of the results. By applying a stringent energy convergence criterion, the model with the most stable energy state was identified as the benchmark for subsequent studies of the ion transport mechanism.

### 3.3. Ion Transport Simulation

In order to deeply investigate the ion transport mechanism in C-S-H gels. The non-reactive force fields currently used for molecular dynamics simulation of cement can be divided into two main categories: generic force fields (e.g., COMPASS) and specific force fields (e.g., ClayFF). Generic force fields have a wide applicability and are able to simulate many different types of molecules and interactions, and the COMPASS force field is particularly suitable for simulating gelling materials because it can accurately calculate the structural-mechanical properties [16]. Therefore, in this paper, molecular dynamics simulations were carried out using the COMPASSII force field to simulate the ion transfer and adsorption processes between the C-S-H gel layers. Ion transfer was focused on the diffusion pattern of corrosive ions in the micropores of the C-S-H gel.

In the simulations, the atomic coordinates of the C-S-H gel fraction, excluding Ca^2+^, were fixed to ensure the stability of the gel structure. The NVT ensemble was employed to maintain the system temperature at 298 K, utilizing the Nose method for precise temperature control. For interatomic interactions, an atom-based electrostatic force treatment was selected, and the Ewald summation method was applied to accurately compute the van der Waals forces. The simulation duration was established at 50 ps, with a step size of 1 fs. The impact of experimental duration on ion adsorption and inter-ion interactions was investigated through the analysis of relative concentration, mean-square displacement, and other relevant parameters.

## 4. Analysis of Results

Usually, cementitious materials, such as cement paste and concrete, possess porous properties, and their pore transport properties have a crucial influence on the overall mechanical properties of the materials. As the main product of cement hydration, C-S-H usually accounts for 60–70% of the whole hydration product and plays an important role in the durability of concrete structures [18].

### 4.1. Analysis of SEM Experimental Results

${\mathrm{SO}}_{4}^{2-}$ can react with calcium hydroxide (Ca(OH)_2_) and C-S-H phases in cementitious materials. This leads to the formation of secondary products such as ettringite (AFt), gypsum (CaSO_4_ 2H_2_O) and other secondary products, which can lead to expansion and deterioration of the material.

**Figure 6:**
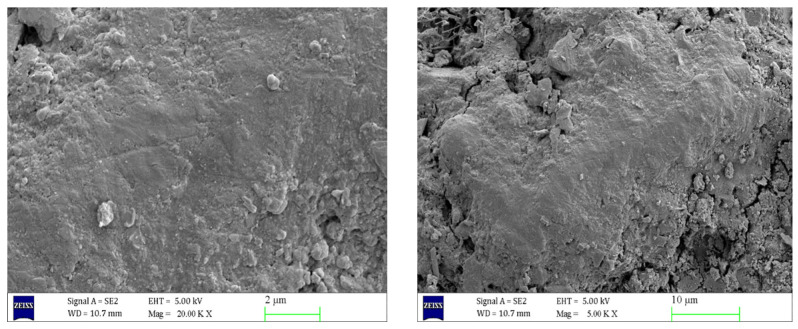
SEM images of concrete before erosion at different magnifications.

**Figure 7:**
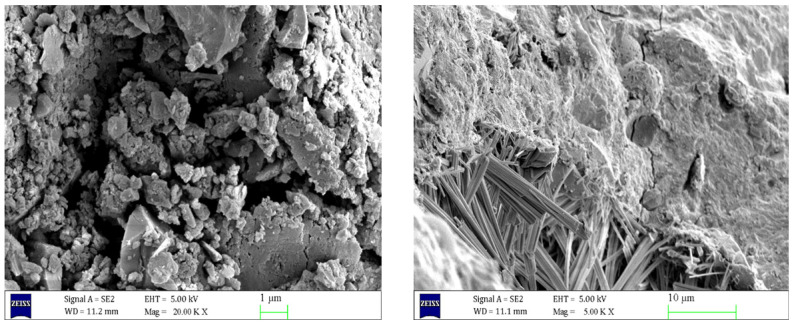
Scanning electron microscope images of 5% sodium sulphate eroded concrete for 90 days at different magnifications.

**Figure 8:**
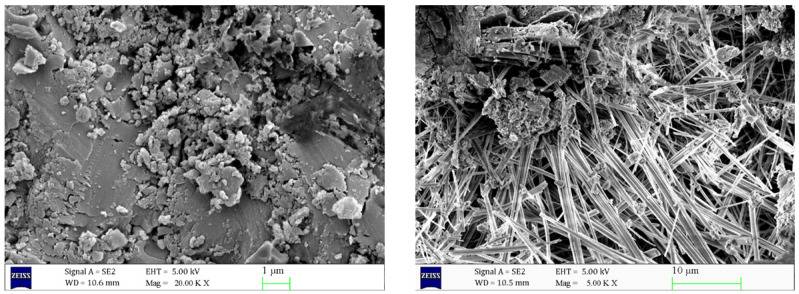
Scanning electron microscope images of concrete after 90 days of 8% sodium sulphate erosion at different magnifications.

As can be seen from Figure 6, inside the uneroded concrete, the C-S-H gel and AFt were uniformly distributed and filled in the concrete matrix, resulting in a tight and neat internal structure of the concrete, with tight connections between the aggregates, and the cracks were almost invisible. However, as can be seen in Figure 7, the structural morphology of the concrete specimens eroded in 5% Na_2_SO_4_ solution for 90 days changed significantly. A large number of needle-like or rod-like AFt were generated at the crack section, and the volume of AFt increased significantly. Figure 8 shows that with the increase of Na_2_SO_4_ solution concentration, Ca(OH)_2_ crystals gradually disappeared, the C-S-H gel became loose, and the number of cracks increased. The volume and number of AFt also further increased, and a large number of needle- or rod-shaped AFt intertwined and overlapped with each other, filling the inside of the cracks.

### 4.2. Changing Law of Relative Concentration of C-S-H/Na_2_SO_4_ Erosion Interface System

**Figure 9:**
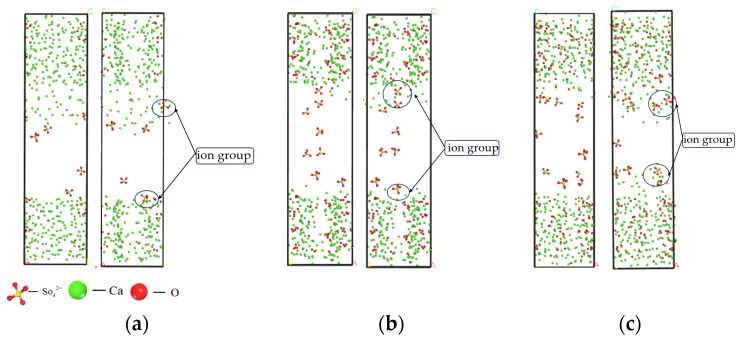
Calcium and Sulfate Ion Distribution at Initial (0 ps) and (50 ps) Time Points. (**a**) Concentration of 5%; (**b**) Concentration of 8%; (**c**) Concentration of 10%.

Since the solid–liquid model was established along the Z direction, the distribution of the relative concentration function in the [0 0 1] direction was selected for calculation. As illustrated in Figure 9, the atomic concentration distribution observed in the vertical direction of the nanoporous substrate demonstrates an approximate symmetry. A strict energy convergence criterion, specifically a total energy change of less than 1 × 10^−6^ eV/atom per iteration, is used to ensure the high accuracy of the computational results.

**Figure 10:**
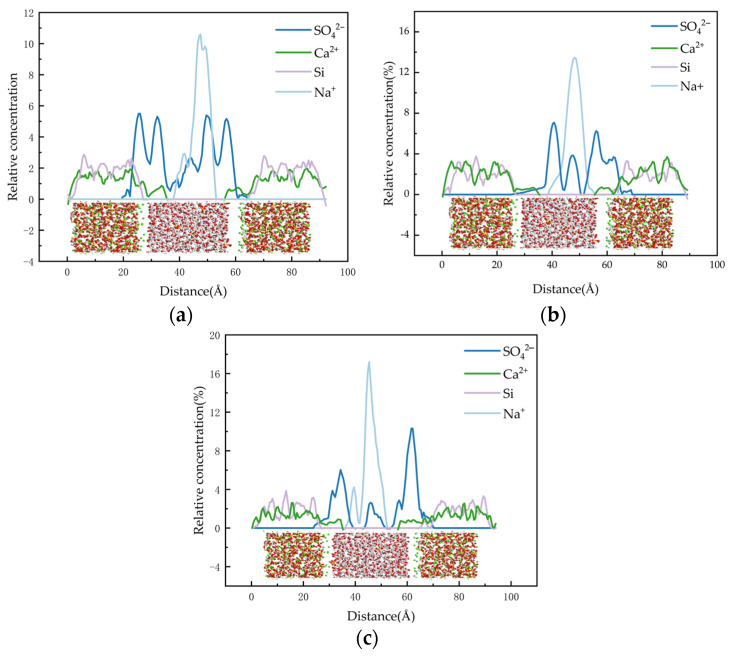
Relative concentrations at the C-S-H/sodium sulfate interface. (**a**) Concentration of 5%; (**b**) Concentration of 8%; (**c**) Concentration of 10%.

**Figure 11:**
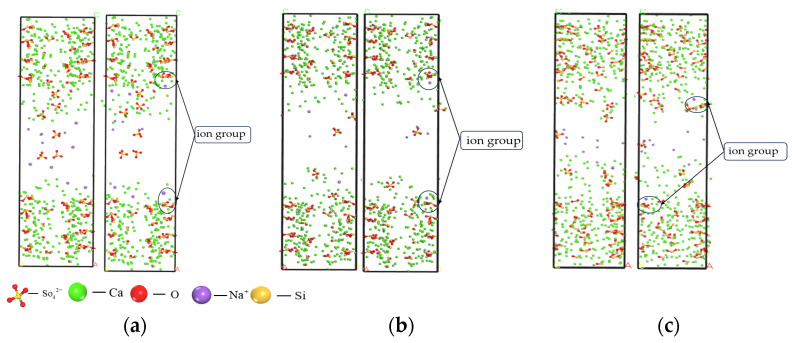
Distribution of sodium and silicon ions at initial (0 ps) and (50 ps) time points. (**a**) Concentration of 5%; (**b**) Concentration of 8%; (**c**) Concentration of 10%.

As shown in Figure 9, during this process, Ca^2+^ on the surface gradually dissociated from the C-S-H structure and migrated toward the middle of the interlayer structure, but its arrangement became more disordered. Some of the dissociated Ca^2+^ was further released into the pore structure and subsequently adsorbed by ${\mathrm{SO}}_{4}^{2-}$ As shown in Figure 10, the relative concentration of ${\mathrm{SO}}_{4}^{2-}$ increased significantly in the range of 20–60 Å, whereas the relative concentration of Na^+^ reached a maximum in the range of 35–45 Å. As can be seen in Figure 11, it is indicated that the pore surfaces may have a stronger adsorption of Na^+^, resulting in a stronger adsorption of Na^+^ concentration increase in this region. Some of the Na^+^ may be competitively adsorbed or ion-exchanged with Ca^2+^ near the silica-oxygen bond. In addition, a small amount of Na^+^ may interact electrostatically with silicate chains to form a certain degree of bonding. Meanwhile, the appearance of strong multi-Si peaks in the C-S-H structure on both sides of the nanopore indicates that there is an enrichment of Si elements in this region.

It is noteworthy that the peaks in the range of 30~35 Å and 50~55 Å show a significant decrease/increase, which indicates that the number of molecules in these regions is relatively small. This phenomenon can be attributed to the presence of nanogaps or the influence of boundary effects during the simulation.

The adsorption of ions on the pore surface of the C-S-H structure showed an enhanced trend with the increase of Na_2_SO_4_ concentration, which indicated that the influence of each atom on the pore surface of the C-S-H structure became more significant.

### 4.3. Radial Distribution Function and Coordination Number of the C-S-H/Na_2_SO_4_ Erosion Interfacial System

In this paper, the Radial Distribution Function (RDF) is used to characterize the unique properties of the C-S-H microstructure as well as the intrinsic laws of spatial arrangement between particles. The RDF between solutions and crystals tends to exhibit significant sharp peak characteristics. The computational expression for the RDF is [19].

(1) $$g(r)=\frac{dN}{4\pi r^{2}\rho dr}$$

where r denotes the distance between particles, N denotes the number of particles, and *ρ* denotes the average density of the system.

The number of nearest neighbor atoms is the coordination number (CN) and is expressed as [4]:

(2) $$CN(r)=\int_{0}^{r}4\pi r^{2}\rho g(r)dr$$

**Figure 12:**
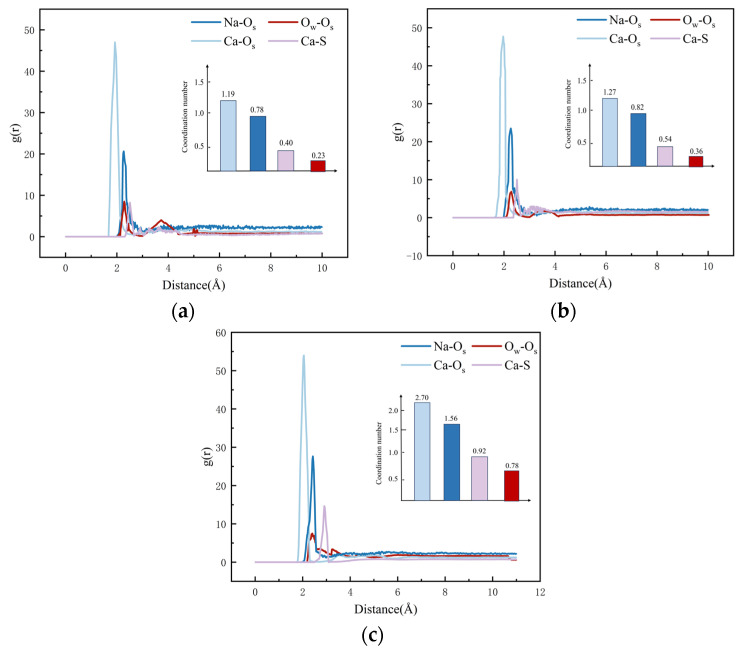
Radial distribution function and average coordination number at different concentrations. (**a**) Concentration of 5%; (**b**) Concentration of 8%; (**c**) Concentration of 10%.

The RDF and average coordination number (CN) of various ions and atoms within the nanopores of C-S-H gels at different concentrations of Na_2_SO_4_ solution are given in Figure 12.

RDF calculations of Na^+^ interacting with non-bridging oxygen atoms (Os) in the silicate chain reveal a distinct peak at a distance of 2.21 Å. As the concentration of the Na_2_SO_4_ solution rises, the intensity of the RDF peak at this distance becomes increasingly pronounced. Notably, the peak RDF intensity of the Na-Os bond reaches 20.63 at a 5% concentration of Na_2_SO_4_, increases to 23.49 at 8%, and further rises to 27.62 at 10%. These changes represent increases of 13.86% and 17.58% compared to the 5% and 8% concentrations, respectively. Importantly, the coordination number of Na-O_s_ bonds achieves its maximum at a 10% concentration, indicating that the strength of the Na-O_s_ bond increases with higher Na_2_SO_4_ concentrations. Additionally, the spatial correlation between Na^+^ and O_s_ at 2.21 Å is significantly enhanced, suggesting that under high-concentration conditions, there are certain interactions between Na^+^ and silicate chains, and some of the Na^+^ binds to the silicate chains through direct bonding (Na-Os) or indirect interactions.

As shown in Figure 12, the RDF calculations of the oxygen atoms Ow and O_s_ in the water molecule show that the two exhibit a strong spatial correlation at about 2.29 Å, and this correlation is not very significantly affected by the Na_2_SO_4_ solution. The spatial correlation of O_w_-O_s_ bonds at 2.42 Å shows a slow weakening trend with the gradual increase of Na_2_SO_4_ solution concentration in the model. In addition, the relatively low coordination number of O_w_-O_s_ suggests that the interaction between the two is weak. This phenomenon suggests that the oxygen coordination number in the hydrogel around the oxygen atoms in the water molecules will be lower; the adsorption capacity of the water molecules with the pore surface of the C-S-H structure is gradually weakened with the increase of the concentration of Na_2_SO_4_ solution. The reason for this is that when the concentration of Na_2_SO_4_ solution increases, more Na^+^ will combine with O_s_ in the silicate chain to form Na-O_s_ bonds, occupying the positions of the water molecules with the non-bridging oxygen atoms in O_w_-O_s_ bonds.

The RDF calculations for Ca^2+^ and O_s_ reveal a pronounced ionic bond peak at approximately 1.97 Å, indicating the strong adsorption capacity of the C-S-H structure for Ca^2+^. Notably, the strength of this adsorption is slightly enhanced with increasing Na_2_SO_4_ concentration. Specifically, the peak RDF strength of the Ca-O_s_ bond was measured at 46.98 for a Na_2_SO_4_ solution concentration of 5%. When the concentration was raised to 8%, the peak strength increased marginally to 47.71, and at a concentration of 10%, it significantly rose to 53.97. This represents increases of 1.55% and 13.12% compared to the 5% and 8% concentrations, respectively. These findings strongly support the notion of robust Ca^2+^ adsorption by the C-S-H structure, with a slight enhancement observed as Na_2_SO_4_ concentration increases.

**Figure 13:**
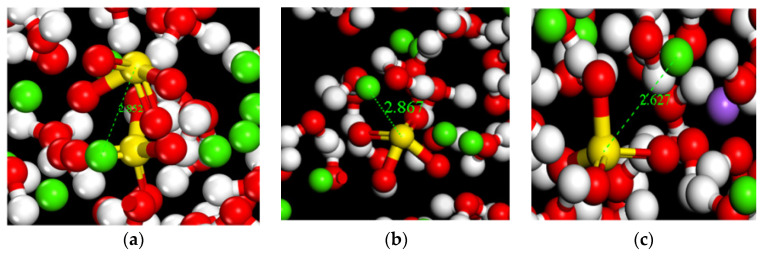
Real-time screenshot at pore surface (green for Ca^2+^, yellow for sulphur atoms, red for O, purple for Na^+^). (**a**) Concentration of 5%; (**b**) Concentration of 8%; (**c**) Concentration of 10%.

RDF calculations of Ca^2+^ in the C-S-H structure and ${\mathrm{SO}}_{4}^{2-}$ (S) in solution reveal that the Ca-S radial distribution function exhibits a strong spatial correlation at 2.51 Å, accompanied by a high peak intensity. Combined with real-time screenshots during the simulation (shown in Figure 13), it can be clearly observed that ${\mathrm{SO}}_{4}^{2-}$ forms ion pairs in the gel nanopores. Further, the peak intensity of Ca-S bonds increased with the gradual increase of ${\mathrm{SO}}_{4}^{2-}$ solution concentration. At a ${\mathrm{SO}}_{4}^{2-}$ solution concentration of 5%, the peak RDF intensity of Ca-S bonds was 8.23; when the concentration was elevated to 8%, the peak intensity increased to 10.02; and at a concentration of 10%, the peak intensity increased significantly to 14.64, which is 17.86% and 46.10% compared to the concentrations of 5% and 8%, respectively. These results indicate that the increase in the concentration of ${\mathrm{SO}}_{4}^{2-}$ solution has a significant facilitating effect on the formation of ion pairs between ${\mathrm{SO}}_{4}^{2-}$ and Ca^2+^ in the silicate chains in the pores of the C-S-H structure. The formation of such ionic clusters may have two effects: on the one hand, it may accelerate the decrease in durability of concrete structures because the formation of ionic clusters may change the microstructure of the material; on the other hand, it may also have a certain hindering effect on the diffusion of Ca^2+^ because the ionic clusters may occupy the space of the pore channels, thus restricting the free movement of ions.

By examining the radial distribution function (RDF) profiles of each chemical bond within the pores of the C-S-H structure at varying concentrations of Na_2_SO_4_, it is evident that the peak strength of the Na-O_s_ bond exhibits a pronounced increasing trend with the gradual rise in Na_2_SO_4_ concentration. These results suggest that the adsorption capacity between Na^+^ and O_s_ is directly influenced by the concentration of Na_2_SO_4_; specifically, higher concentrations of Na_2_SO_4_ correlate with a stronger interaction observed in the Na-O_s_ bonds within the RDF profiles. This implies that Na^+^ can adsorb more stably onto the silicate chains as the concentration of Na_2_SO_4_ increases.

Conversely, as the concentration of Na_2_SO_4_ increases, the peak strength of the O_w_-O_s_ bond, formed by the interaction of water molecules with O_s_ in solution, exhibits a slight decreasing trend. This observation suggests that higher concentrations of Na_2_SO_4_ weaken the adsorption of water molecules onto the C-S-H surface. The primary reason for this phenomenon is that, with increasing Na_2_SO_4_ concentration, a greater number of Na^+^ tend to bind to O_s_, resulting in the formation of Na-O_s_ bonds that occupy the binding sites of the O_w_-O_s_ bonds.

With the gradual increase in the concentration of Na_2_SO_4_ solution, the RDF peak strength of the Ca-S bond formed between ${\mathrm{SO}}_{4}^{2-}$ and Ca^2+^ also showed an enhanced trend. This phenomenon suggests that the increase of Na_2_SO_4_ concentration promotes the adsorption interaction between ${\mathrm{SO}}_{4}^{2-}$ and Ca^2+^ in the C-S-H structure. At the same time, ${\mathrm{SO}}_{4}^{2-}$ and Ca^2+^, as a ion carrying opposite charges, was closely linked by electrostatic attraction to form a stable ionic cluster structure. This dynamic process may gradually lead to the deterioration of the C-S-H gel structure, which is observed at the macroscopic level and is manifested by the damage phenomena such as the increase of pores and the extension of cracks within the concrete structure. These structural changes further exacerbate the penetration and diffusion of ${\mathrm{SO}}_{4}^{2-}$ into the cementitious materials, which ultimately triggers a significant decline in the durability of concrete structures.

### 4.4. Mean-Square Displacements and Diffusion Coefficients for the C-S-H/Na_2_SO_4_ Erosion Interface System

Atoms gradually tend to reach a relatively uniform distribution in the spatial domain of the simulation through a continuous diffusion and vibration mechanism. In the MD simulation framework, the positions of the atoms are not fixed but exhibit random motions as the simulation progresses, and their motion rates are modulated by both the ambient temperature and the microstructure of the material. The mean-square displacement (MSD) is used as an important metric to quantitatively describe the time-dependent displacement behavior of atoms in microscopic systems with the expression [20]:

(3) $$\mathrm{MSD}(\Delta t)=\frac{1}{T-\Delta t}{\int_{0}^{T-\Delta t}\left[r(t-\Delta t)-r(t)\right]}^{2}dt=\langle {\left|r_{i}(t)-\left.r_{i}(0)\right|\right.}^{2}\rangle$$

where *r_i_*(*t*) and *r_i_*(0) are the position vectors of atom *i* at times *t* and 0, respectively, and < > denotes the overall average value.

There is a one-to-one correspondence between the amount of MSD of a calculated atom and its diffusion coefficient. Although the diffusion coefficient of the trajectory of the studied system cannot be found directly, the diffusion coefficient of the target system can be found indirectly by calculating the slope of the MSD; i.e., the diffusion coefficient is one-sixth of the slope of the MSD curve. The diffusion coefficient (diffusion coefficient) can represent the transport properties of the atoms in the system and compare the movement rates of various particles; the expression is [10]:

(4) $$D_{a}=\frac{1}{6}\underset{t\to \infty }{\lim}\frac{dMSD}{dt}=\frac{1}{6N_{\alpha }}\underset{t\to \infty }{\lim}\frac{d}{dt}\sum_{t=1}^{Na}\langle \left[r_{i}(t)-r_{0}(t)\right]\rangle$$

where $r_{i}(t)$ is the position vector of particle *i* at time t; $r_{0}(t)$ is the position vector of particle *i* at the initial time; N is the number of diffusing atoms in the system.

**Figure 14:**
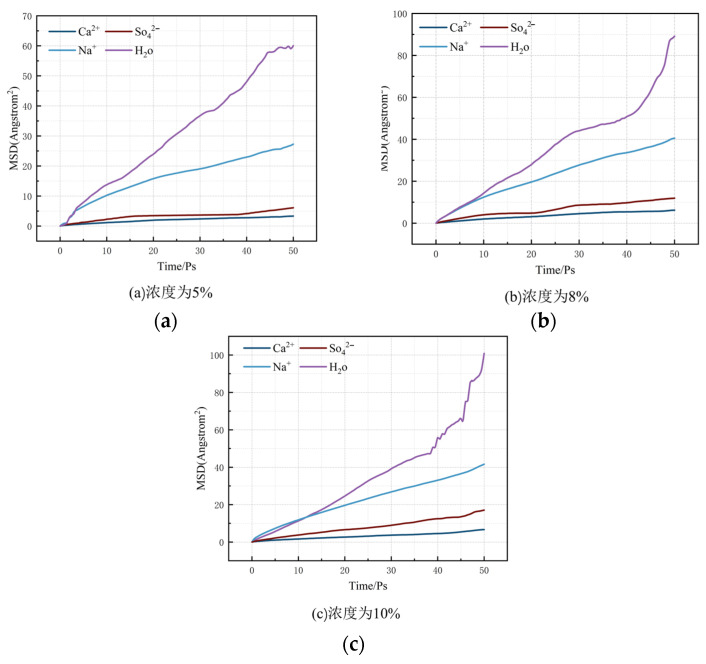
MSD at different concentrations. (**a**) Concentration of 5%; (**b**) Concentration of 8%; (**c**) Concentration of 10%.

Figure 14 demonstrates the MSD curves of water molecules, ${\mathrm{SO}}_{4}^{2-}$, Na^+^ and Ca^2+^ in the C-S-H structure at different Na_2_SO_4_ solution concentrations.

The results indicated that the mean square displacement (MSD) curves of water molecules were significantly higher than those of ${\mathrm{SO}}_{4}^{2-}$, Na^+^ and Ca^2+^. This reflects the rapid diffusion behavior of water molecules in solution. The relatively low MSD of ${\mathrm{SO}}_{4}^{2-}$ implies that its movement is relatively restricted, but its interaction with water molecules may cause it to exhibit co-migration with water molecules in aqueous solution. The higher MSD of water molecules indicates their stronger free movement, while the stronger interaction of ${\mathrm{SO}}_{4}^{2-}$ ions with water molecules may result in the faster migration of ${\mathrm{SO}}_{4}^{2-}$ ions with water. Therefore, ${\mathrm{SO}}_{4}^{2-}$ does not migrate alone, but together with water molecules, and thus exhibits “osmotic” properties more rapidly than other ions. Notably, the diffusion rate of water molecules within the pore channels of the C-S-H structure is characterized by relatively high speed. This phenomenon can be attributed to the influence of the potential energy of the pore walls on both the water molecules and ${\mathrm{SO}}_{4}^{2-}$ within the pore structure, which affects the diffusion coefficients of the internal particles. As the concentration of the solution increases, the potential energy exerted by the pore walls on the internal particles gradually diminishes, leading to a corresponding increase in the diffusion coefficients of these particles.

In summary, as the concentration of Na_2_SO_4_ solution increases, the offset of Ca^2+^ exhibits a progressively increasing trend. This migration may affect the structural stability of concrete matrices composed mainly of calcium silicate hydrates (C-S-H); this phenomenon is reflected in the surface defects of cement hydration products, including characteristics such as honeycomb surfaces and pit surfaces, which are accompanied by a gradual decrease in the material’s strength.

Table 2 shows the diffusion coefficients of water molecules and each ion at each concentration.

According to the data analysis presented in Table 2, the diffusion coefficient (D_a_) of ${\mathrm{SO}}_{4}^{2-}$ in each nanopore size within the model exhibited an increasing trend with the gradual increase in the concentration of Na_2_SO_4_ solution. Specifically, at a 5% Na_2_SO_4_ concentration, the diffusion coefficient Da was measured at 0.33 × 10^−9^ m^2^/s. As the concentration of Na_2_SO_4_ rose to 8% and 10%, the diffusion coefficient Da values of ${\mathrm{SO}}_{4}^{2-}$ increased to 0.37 × 10^−9^ m^2^/s and 1.12 × 10^−9^ m^2^/s, respectively. This phenomenon suggests that higher concentrations of Na_2_SO_4_ solution effectively facilitate the diffusion of ${\mathrm{SO}}_{4}^{2-}$ into the hydrogel, thereby enhancing the driving force for ${\mathrm{SO}}_{4}^{2-}$ osmosis. As ${\mathrm{SO}}_{4}^{2-}$ gradually enters the hydrogel, they undergo a displacement reaction with some Ca^2+^ in the gel, resulting in the precipitation of some Ca^2+^ from the hydrogel structure. This series of reaction processes accelerated the erosion and degradation of the hydrogel, which in turn adversely affected its structure and performance.

## 5. Discussion

In this study, we focused on the erosive effect of different concentrations of sodium sulfate (Na_2_SO_4_) on concrete and accordingly evaluated its effect on concrete durability. However, it is worth noting that the pH of the solution may be an important influencing factor in practical situations, which may significantly affect the reaction path and rate of sodium sulfate with concrete components.

However, during the experimental design and implementation phase of this study, we chose to focus on the primary variable of sodium sulfate concentration to more clearly reveal its effect on concrete performance. Nonetheless, we recognize that integrating multiple factors, including pH, would be a valuable direction to take in future studies. By simulating sodium sulfate erosion experiments under different pH conditions, we can gain a more comprehensive understanding of the durability of concrete in sulfate environments and provide more accurate guidance for practical engineering applications.

## 6. Conclusions

In this paper, a molecular dynamics approach was used to investigate the microscopic mechanism of C-S-H gel erosion in sulfate environments, with the following conclusions:

With the gradual increase in Na_2_SO_4_ concentration, the electrostatic adsorption interaction between Na_2_SO_4_ and Ca^2+^ in the C-S-H structure was significantly enhanced, resulting in an upward trend in the peak strength of the radial distribution function (RDF) of Ca-S bonds. Furthermore, the increase in the number of pores and cracks created more accessible pathways for the diffusion of ${\mathrm{SO}}_{4}^{2-}$ into the interior of the concrete, thereby accelerating the deterioration of concrete durability.

The introduction of ${\mathrm{SO}}_{4}^{2-}$ not only promoted its own diffusion in the concrete but also the small-scale ionic clusters formed by Ca^2+^ and ${\mathrm{SO}}_{4}^{2-}$ in the local area accelerated the migration of Na^+^ and Ca^2+^, further aggravated the erosion effect, and seriously affected the overall performance of the concrete; the pore surfaces of the C-S-H structure had a significant adsorption capacity for Na^+^. With the increase of Na_2_SO_4_ solution concentration, Na^+^ is more easily adsorbed firmly on the pore surface through the formation of Na-O_s_ bonds. When the adsorbed Na^+^ on the C-S-H pore surface accumulates to a certain concentration, it will act as a charge center, inducing ${\mathrm{SO}}_{4}^{2-}$ to migrate towards the pore wall gradually and slowly.

With the increasing concentration of Na_2_SO_4_ solution, the adsorption capacity of ions on the surface of the C-S-H structure increases. A large amount of Na^+^ will be retained on the pore surface of C-S-H, and the attraction of ${\mathrm{SO}}_{4}^{2-}$ in the solution increases accordingly. Therefore, ${\mathrm{SO}}_{4}^{2-}$ and Ca^2+^ in the C-S-H structure is more likely to form ionic clusters, resulting in a significant decrease in the mechanical properties of the C-S-H structure. Once a large number of cracks appeared in the concrete structure, ${\mathrm{SO}}_{4}^{2-}$ would be more likely to penetrate into the cement paste, which would further aggravate the deterioration process of the structure.

## Figures and Tables

**Table 1:** Parameters of the C-S-H/Na_2_SO_4_ model.

Mass Fraction Concentration	Water Molecule Number	Sulfate Number	Total Number of Particles	Model Size/Å
5%	630	5	3396	22.11 × 22.68 × 92.46
8%	608	8	3265	
10%	540	9	3534	

**Table 2:** Diffusion coefficients of water molecules and ions at various concentrations.

Mass Fraction Concentration	Water Molecule Diffusion Coefficient/(×10^−9^ m^2^/s)	Na^+^ (×10^−9^ m^2^/s)	SO_4_^2−^ (×10^−9^ m^2^/s)	Ca^2+^ (×10^−9^ m^2^/s)
5%	1.54	0.92	0.33	0.17
8%	1.78	1.17	0.37	0.16
10%	1.84	1.64	1.12	0.09

## Data Availability

The original contributions presented in this study are included in the article. Further inquiries can be directed to the corresponding author.
